# Preventing Disruptions in HIV Service Delivery to Key Populations During Project Transition From an International to a Local Implementing Partner: A Case Study From Zambia

**DOI:** 10.9745/GHSP-D-24-00186

**Published:** 2025-12-31

**Authors:** Edward Adekola Oladele, Maurice Musheke, Florence Mulenga, Alick Samona, Ihoghosa Iyamu, Arlene Phiri, Ngaitila Phiri, Otto N. Chabikuli

**Affiliations:** a FHI 360 Zambia, Lusaka, Zambia. Now with Cutting Edge Public Health Solutions.; b Centre for Infectious Disease Research in Zambia, Lusaka, Zambia.; c FHI 360 Zambia, Lusaka, Zambia. Now an independent consultant.; d Centre for Infectious Disease Research in Zambia, Lusaka, Zambia. Now an independent consultant.; e School of Population and Public Health, University of British Columbia, Vancouver, Canada.; f U.S. Agency for International Development Zambia, Lusaka, Zambia. Now an independent consultant.; g FHI 360, Eastern and Southern Africa Regional Office, Pretoria, South Africa. Now with the Centre for Infectious Disease Research in Zambia.

## Abstract

In the management of chronic conditions like HIV, the continuity of service delivery is necessary to achieve desired outcomes, such as HIV viral load suppression, behavioral change, improved health, and client satisfaction. The transition phase—when a project closes and another starts—is a potential period of service delivery disruption. Active management of this transition period is important to prevent disruptions, especially for key populations who may be stigmatized and have limited options for accessing HIV services. We analyzed this transition period between July and December 2022 between 2 projects that provided HIV prevention services, management of sexually transmitted infections, and linkage to HIV treatment and other complementary services to key populations in Zambia. To ensure a smooth project transition, we implemented a set of interventions, including joint planning for project transition, strategic leadership, trust-building initiatives, active community and stakeholder engagement, repeated stakeholder reassurance, open communication, and transparent data sharing. After transitioning to the new project, we noted that all 3 service types of interest experienced at least a 20% increase over the levels achieved in the last month of the closing project. This increase contrasts with the assumption that all service types delivered through project structures would decline to zero persons reached within 2 months of project closing if the next project did not commence seamlessly. The decrease in service delivery was averted with the intentional transition interventions. Additionally, we recorded operational gains, such as stakeholder satisfaction, adequate assets transfer, stability in project service delivery location, and reduced personnel anxiety. We conclude that active multipartite management of the transition phase for projects is essential for ensuring uninterrupted service delivery and sustaining good outcomes for clients. Donors, health system managers, and program managers should actively require and design sound transition management plans as part of their program designs. In the aftermath of recent abrupt cuts in US Government development sector funding that allowed no planned transitions, it is important that surviving programs carefully imbibe lessons shared in this paper to protect years—and sometimes decades—of program gains.

## Abstract (precis)

Preventing interruption in service delivery during project transition is essential to sustain good client outcomes. Adequate planning, strategic leadership, trust-building, active community engagement, repeated stakeholder reassurance, open communication, and transparent data sharing are key elements of a well-managed project transition to ensure uninterrupted service delivery.

## Teaser

**Key Findings:**

- Uninterrupted service delivery during the transition between 2 donor-funded projects managed by different organizations but serving the same community was feasible by implementing strategic leadership that set a vision and engaged all stakeholders.
- A successful transition of services occurred because all aspects of project management, including meaningful inclusion of stakeholders, community engagement, staffing, service delivery location, physical assets, and data management, were intentionally managed.
- Clear communication from all parties involved created trust in the program managers, prevented conflicting messages, and enhanced stakeholder support for the new project and recognition of the outgoing project.

**Key Implications:**

- Program managers should adequately plan for and resource the transition phase when designing and managing projects.
- Donors and national health system managers should require implementers to describe specific transition phase management approaches in their overall project management plans.

## INTRODUCTION

Uninterrupted service delivery, especially within programs focused on chronic conditions, is essential to support clients and is strongly associated with good health outcomes.^1^^–^^3^ Indeed, many practitioners and researchers have established that a high level of continuity of care is associated with reduced mortality,^4^ HIV viral suppression,^5^ fewer hospitalizations,^6^ lower health care expenses,^7^^,^^8^ improved medication adherence, and higher client satisfaction.^9^ By nature, donor-funded programs run on a cycle—projects end while new (follow-on) ones start. With greater documentation and understanding of the importance of continuity of care, funders desire minimal service delivery disruption during the transition between closing and follow-on projects. Implementers also often commit in their proposals to minimize disruptions in service delivery during transitions. Planning for this transition is even more important when the organizations managing the closing and starting projects are different and, as in our case, when transitioning from an international to a local partner.

In our experience, we have observed multiple instances of how funders’ desires and implementers’ promises of minimal service disruptions never materialize. Communities often experience decreased service intensity before it rebounds when the new project stabilizes.^10^ For stigmatized populations whose options for services are usually limited to project-supported services specifically designed for them, active transition management to ensure continuity of services becomes even more important. Gotsadze and colleagues noted that the public health gains from successful projects can be at risk if transitions are poorly planned and executed.^11^ Indeed, a poor transition can set back service delivery by as long as a year—the time it may take a new project to settle in. Abrupt cuts that allow no transition have even worse consequences, as evidence from the impact of the January 2025 US Government Stop Work Orders shows.^12^^,^^13^

As the U.S. Agency for International Development (USAID) was implementing its localization agenda,^14^^,^^15^ progress was being made on many fronts, albeit slowly. One of its pillars, direct funding to local partners, aimed to channel a significant portion of funding directly to local organizations while ensuring accountability, with slow but steady progress.^16^ Over the last 5 years before USAID was dissolved, USAID had seen an uptrend marked by significant increases in direct local funding, reaching a high of nearly US$1.6 billion (10.2%) in fiscal year 2022 (October 1, 2022, to September 30, 2023). USAID missions in Africa had been particularly noteworthy in expanding work with local partners, with nearly 25% of their funding going to local partners during this period.^17^ This progress had led to mixed results (impact on communities) and mixed responses from the development community.^18^

USAID had funded FHI 360, an international nongovernmental organization (NGO), to lead the USAID Open Doors project implementation in Zambia.^19^ In line with USAID’s localization agenda, before the project closeout in December 2022, in October 2021, USAID awarded the follow-on project, USAID Controlling HIV Epidemic for Key and Underserved Populations (CHEKUP) I project,^20^ to the Centre for Infectious Disease Research in Zambia (CIDRZ), a local NGO. Leading up to the USAID Open Doors project’s closeout date, teams from both projects and USAID Zambia collaborated closely to ensure a smooth transition.

In this article, we discuss the transition between the projects, share the key elements of that transition, and describe how this model may be useful for transitions between closing and commencing projects and between international and local partners. Despite the dire consequences of poorly implemented transitions on client outcomes, there is paucity of published literature focusing on transition of donor-funded projects from one implementing partner to another. Literature focusing on transition between 2 donor-funded projects from an international partner to a local partner in the localization era is even more rare. This article therefore contributes to evidence on what should constitute good transition practice when transitioning between international and local NGOs. Acting on evidence such as what we share in this article could have reshaped the abrupt disruption experienced in February 2025 that had severe implications on people’s lives.^12^

## PROJECT DESCRIPTIONS

The USAID Open Doors project, funded by the U.S. President’s Emergency Plan for AIDS Relief (PEPFAR), was implemented by FHI 360 in 8 districts in Zambia from May 2016 to December 2022. The project provided HIV prevention and related services, including HIV testing and counseling, sexually transmitted infection screening and treatment, HIV pre-exposure prophylaxis (PrEP) and post-exposure prophylaxis, behavior change communication, risk reduction interventions, family planning, economic empowerment, mental health as well as initiation of antiretroviral therapy (ART), to those at heightened risk of acquiring or transmitting HIV infection, called key populations (KPs).^21^^,^^22^ The project also provided referrals of clients for management of advanced HIV disease where necessary, and referrals to other social services not directly offered by the project (e.g., legal services). The project was hosted by the Ministry of Health and worked closely with line government ministries (e.g., justice and gender) and internal security agencies, such as the Zambia police service. The project implementation applied the principle of having greater involvement of people living with HIV^23^ and subgranted KP-led and KP-competent organizations to directly implement the project’s services. The KP community in Zambia is often discriminated against and highly stigmatized.^24^ Laws are often misinterpreted to the disadvantage of the community, and public backlash against KPs often happens in cycles that are sometimes fueled by political actors. Organizations providing KP services are also sometimes targeted for attacks during periods of public backlash against the KP community. Therefore, providing services for KPs in Zambia is often challenging and must be responsive to the needs of the KP community. Consequently, implementation arrangements are sometimes through project-funded structures until, through dedicated engagement and capacity-building, the services can be taken over directly by the government.

The USAID Open Doors project established wellness centers, identified in collaboration with the KP community to ensure safety and confidentiality, in each of the districts for service provision and support activities. Synergies were established with treatment partners and the Ministry of Health (represented at the district level by district health management teams) for the supply of program commodities (e.g., HIV rapid test kits), and provision of additional clinical services (e.g., ART for advanced HIV disease, and voluntary medical male circumcision). Each center was annexed to a named government health facility that served as the hub for supplying health commodities to the wellness centers. The hub facility also served as a referral center for managing complex cases beyond the wellness center’s capacity. Each geographical location was divided into zones, and a peer leader managed demand-creation activities in the zones. Within the zones, hot spots were mapped and appropriate services were planned using a decentralized service delivery (differentiated service delivery) model suitable for clients. Wellness centers also operated on flexible schedules to allow KPs to access services when it was convenient for them.

The follow-on USAID CHEKUP I project, implemented by CIDRZ, aimed to improve the health outcomes of Zambians by preventing new infections among both priority populations^25^ and KPs most at risk of acquiring HIV. This combination of KP and priority populations is the first of its kind in Zambia and aims to bridge the service linkages gap for these highly at-risk populations.

The project was awarded in October 2021, but the KP component began in July 2022. The project’s main objectives were to: (1) identify and reach individuals at high risk of HIV infection in communities with high-impact HIV prevention services; (2) use targeted testing strategies to find and link newly identified clients to HIV treatment and support services; (3) build the capacity of communities and leverage community structures to implement evidence-based, high-impact strategies; and (4) build the capacity of local implementing partners to provide targeted, evidence-based HIV prevention services in line with USAID strategies.

For both projects, services to KPs were the same and followed the standard cascade of KP services initially developed by the LINKAGES project but now globally adopted as the standard.^26^^,^^27^ For the analysis in this article, we included 3 service types that were routinely documented in project records (using PEPFAR monitoring, evaluation, and reporting indicators^28^) in the 3 districts (Solwezi, Chililabombwe, and Kitwe) in which both projects had shared responsibility (Box). These service types show the reach of clients through project activities.

BOX Three HIV Service Types Analyzed and Their Components, Zambia

1. Prevention interventions for individuals or small groups (fewer than 25 people) in key populations included:
  - Offering or referring to HIV testing services
  - Providing targeted information, education, and communication
  - Conducting outreach and empowerment activities
  - Distributing or referring to services for condoms and lubricants
  - Offering or referring to sexually transmitted infection screening, prevention, and treatment
  - Linking or referring to antiretroviral therapy services
  - Offering or referring to prevention, diagnosis, and treatment of tuberculosis
  - Offering or referring to screening and vaccination for viral hepatitis
  - Providing or referring to reproductive health services, including family planning and prevention of mother-to-child transmission of HIV, if applicable
2. HIV testing services, including delivery of test results
3. Enrollment on oral antiretroviral pre-exposure prophylaxis to prevent HIV infection

## PROJECT TRANSITION APPROACH

### Conceptual Framework

**FIGURE 1:**
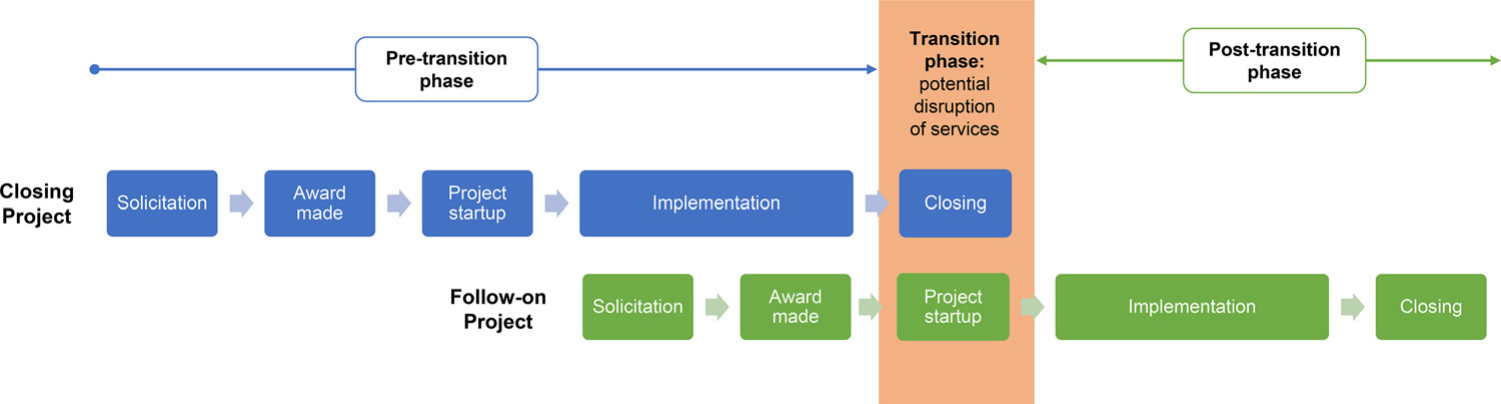
Conceptual Temporal Location of the Transition Phase in the Projects’ Lifespan

In this article, we propose the concept of a “transition phase” in the project lifespan, the time frame when there is a temporal overlap between 1 project ending and another beginning (Figure 1). This transition phase conceptually splits activities into 3 phases: pre-transition, transition, and post-transition phases. It is important to emphasize that this is a temporal concept related to timelines and is best applied to 2 overlapping projects in a temporal sequence. It is also helpful to note that a project’s post-transition also constitutes the pre-transition phase in relation to the follow-on project. Our article focuses on the transition phase and the key elements within that phase that ensured uninterrupted service delivery to clients.

### Key Elements of the Intervention

**FIGURE 2:**
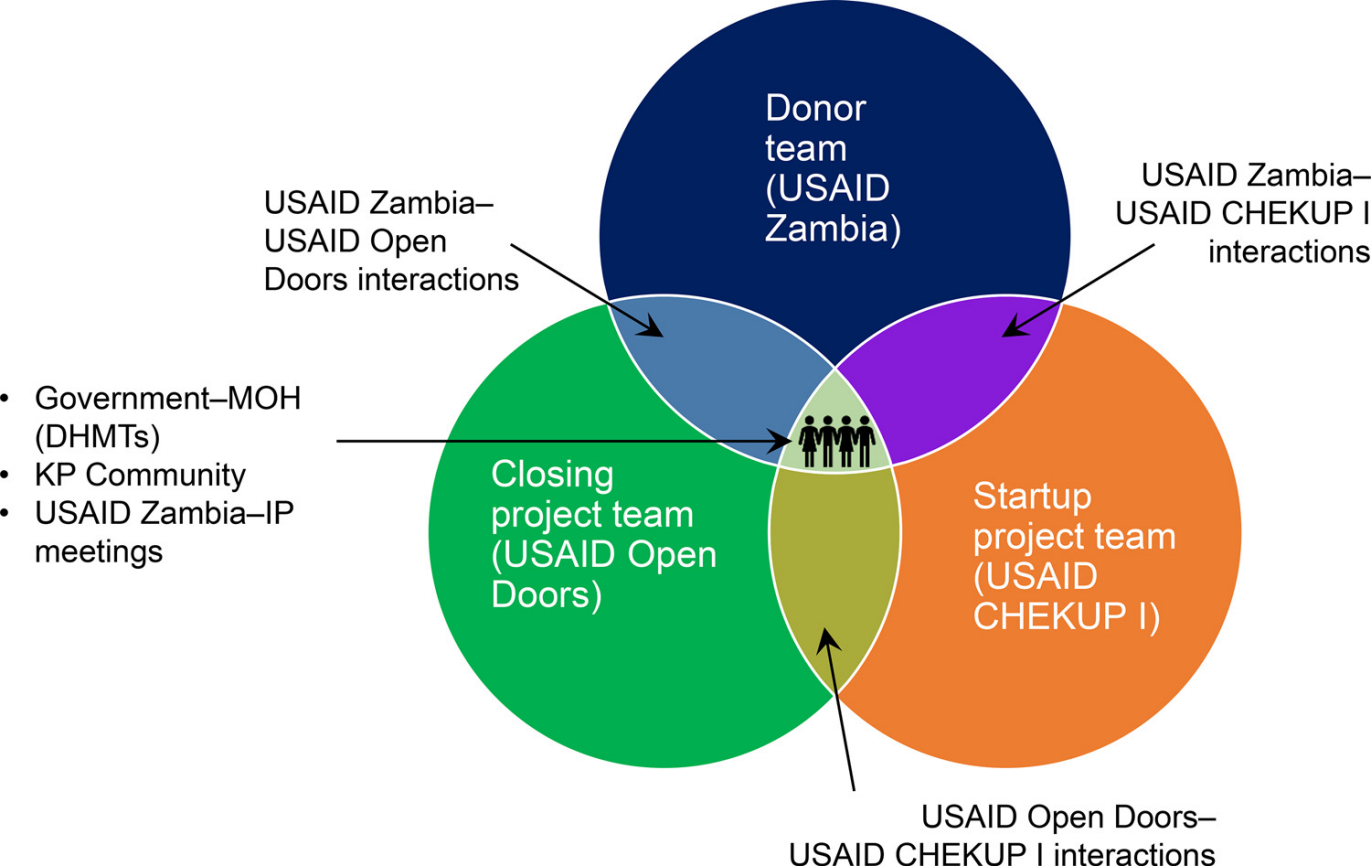
Depiction of the Different Levels of Communication for Smooth Transition Between Two Projects Abbreviations: CHEKUP, Controlling HIV Epidemic for Key and Underserved Populations; DHMT, district health management team; IP, implementing partner; MOH, Ministry of Health; USAID, U.S. Agency for International Development.

To ensure a smooth transition between the 2 projects, both projects adopted a people-centered communication approach (Figure 2) among the project teams and donor team with community engagement at its core, allowing each team to continue to fulfill its mandate. The communication approach had key elements that ensured success: USAID leadership and agenda setting, interaction between implementing partners, active community engagement, stakeholder reassurance, and data sharing. We measured this success in both qualitative outputs and quantitative measures of service delivery numbers.

#### USAID Leadership and Agenda Setting

USAID led the smooth transition by introducing the 2 implementing partners (FHI 360 and CIDRZ) via email and subsequently setting up a meeting where teams shared expectations, discussed the approach, and reached general agreements. USAID also ensured that although the 2 projects had a 3-month overlap, service delivery and data reporting responsibilities were clearly delineated. There was no overlap in the period that each project had accountability for reporting and, therefore, no potential for double reporting. USAID facilitated several partner meetings and included representation from the KP community to ensure their meaningful engagement in the transition process.

#### Interactions Between Implementing Partners and Open Communication

Having been introduced, the 2 implementing partners met frequently to openly discuss concerns, agree on areas of intervention, and set the tone for focused communication. There was a common agreement between the leaders of both projects that (1) both teams would be available to each other to share lessons and potential challenges, as well as communicate openly and frequently; (2) the follow-on project would leverage existing platforms during the start-up period and not reinvent already established systems; and (3) the new project’s start-up period activities would not be at the expense of the service delivery of the closing project.

The third item, derived from the shared commitment to ensure continued service delivery, served as the foundation for a phased transition of human resources, timing of site handovers, and other aspects that could impact service delivery. The transition focused on several key areas: implementing partner staff; service delivery teams (volunteers, peer promoters, outreach workers, counselors, and clinicians); wellness center spaces and facilities; community service delivery hubs; community engagement; and assets transfer, including office equipment, training materials, job aids, information systems, and mechanisms for engaging KP civil society organizations (KP CSOs). USAID Open Doors staff were encouraged to apply for jobs under USAID CHEKUP I to leverage USAID investment in building the capacity of these individuals and ensure that the already gained knowledge in KP programming approaches and interventions were immediately available to provide services to the KP community. To minimize disruption of services delivered by USAID Open Doors in the final weeks, both partners agreed on a schedule for transitioning staff who had successfully interviewed for CHEKUP I to ensure a smooth and quick start-up and continued services to the KP community.

#### Active Community Engagement

In the USAID Open Doors project closeout stage (as well as throughout the project lifecycle), the project team closely engaged KP-led and KP-competent CSOs involved with implementation in the closeout discussions. The USAID CHEKUP I team also adopted an active engagement process to ensure that the CSOs knew of the impending transition and could provide inputs into the shape of the transition, ensuring that the KP community was well prepared for potential changes and could adjust accordingly. This effectively prevented frustrations during the transition process. The USAID Open Doors project facilitated the linkage and engagement of KP-led CSOs with USAID CHEKUP I in program implementation strategies, selection of wellness centers, and, ultimately, the design of requests for applications for contractual engagement with KP-led CSOs. The USAID CHEKUP I team issued initial contracts to keep all volunteers and peer promoters, outreach workers, and staff who were on the USAID Open Doors project to continue service provision in the interim transition period while the project was engaged in full onboarding of KP CSOs. As services to the KP community are often peer-driven through known and trusted workers, this approach ensured that all existing KP networks that were reached with services under the USAID Open Doors project were immediately accessible to the new project with no service disruption. Due to legal restrictions on KPs, especially men who have sex with men in Zambia, KPs often live in fear and uncertainty about their safety. Often, KPs do not openly seek health services. The trusted networks were important in facilitating access to services by KPs and supporting a stigma-free and safe health facility environment.

#### Stakeholder Reassurance

When the solicitation for USAID CHEKUP I was released (pre-transition phase), the USAID Open Doors project informed both internal and external stakeholders that USAID support for the program would not end abruptly but continue under a new project. Once USAID CHEKUP I was awarded, the USAID Open Doors project team informed stakeholders about the successor project. At the district level, the USAID CHEKUP I project team attended USAID Open Doors closeout meetings to be introduced to all local stakeholders, including the Ministry of Health district office and National AIDS Council district and provincial offices, and share the USAID CHEKUP I vision and strategy for continuing services to KPs after closeout. KP CSOs that worked in the districts of operation for the USAID CHEKUP I project attended the district closeout meetings and had an opportunity to state their commitment to work with the new project and the KP community.

#### Data Sharing

After district closeout meetings, the USAID CHEKUP I project was ready to provide services. However, CHEKUP I needed to retain client registers to verify clients’ continuation on HIV and sexual and reproductive health services. To meet donor requirements and international standards for safeguarding client-identifiable data and commitment to confidentiality, specific steps were taken as part of the project transition. First, an agreement/letter of understanding that covered shared access to project data, shared confidentiality, data safety and security, and electronic data sharing was in place to safeguard client data and ensure access to registers by both projects. Second, service delivery data were transitioned to allow the Open Doors project to complete data verification and prepare project final reports while allowing the new project to begin managing data to avoid gaps in serving clients. Finally, data collection tools used for the USAID Open Doors project and shared with CHEKUP I included a KP classification tool, HIV testing services screening tool, service delivery tool, intimate partner violence screening tool, referral form, and registers (e.g., HIV testing services, ART, PrEP, sexually transmitted infection screening, and family planning). All data collected during the USAID Open Doors project were shared over 3 months of the USAID CHEKUP I project start-up period.

## ACTIVE TRANSITION MANAGEMENT OUTPUTS

### Operational Results

To ensure the smooth coordination of program implementation, the USAID CHEKUP I project, in consultation with the USAID Open Doors project, employed key former staff (e.g., 4 site coordinators) from the Open Doors project to manage KP activities in 4 districts of operation (Solwezi, Ndola, Luanshya, and Kitwe). These site coordinators brought their existing relationships with local stakeholders and proved invaluable to the transition process.

Some selected key implementation teams were engaged from CSOs, including health care providers, peer promoters, lay counselors, and outreach workers, to ensure continued mobilization and client confidentiality as clients continued to interact with staff with whom they were familiar.

USAID CHEKUP I project signed new leases for the same KP safe spaces and wellness centers that USAID Open Doors used, which the community had come to know, ensuring that clients did not experience geographic relocation disorientation. The USAID CHEKUP I project updated the branding on the signposts to indicate the new project.

Key service delivery assets, including technical materials, office furniture, and office equipment, were transferred from the outgoing project to the new project to ensure continued service provision.

The KP community demonstrated an understanding of the transition process and patience to allow for transitional processes to happen. KP community stakeholders actively participated in the transition process by giving inputs into the process, informing community hub transitions, contributing to the request for application issued by the new project to KP CSOs, and allaying anxiety among the KP community.

### Service Delivery Results

**FIGURE 3:**
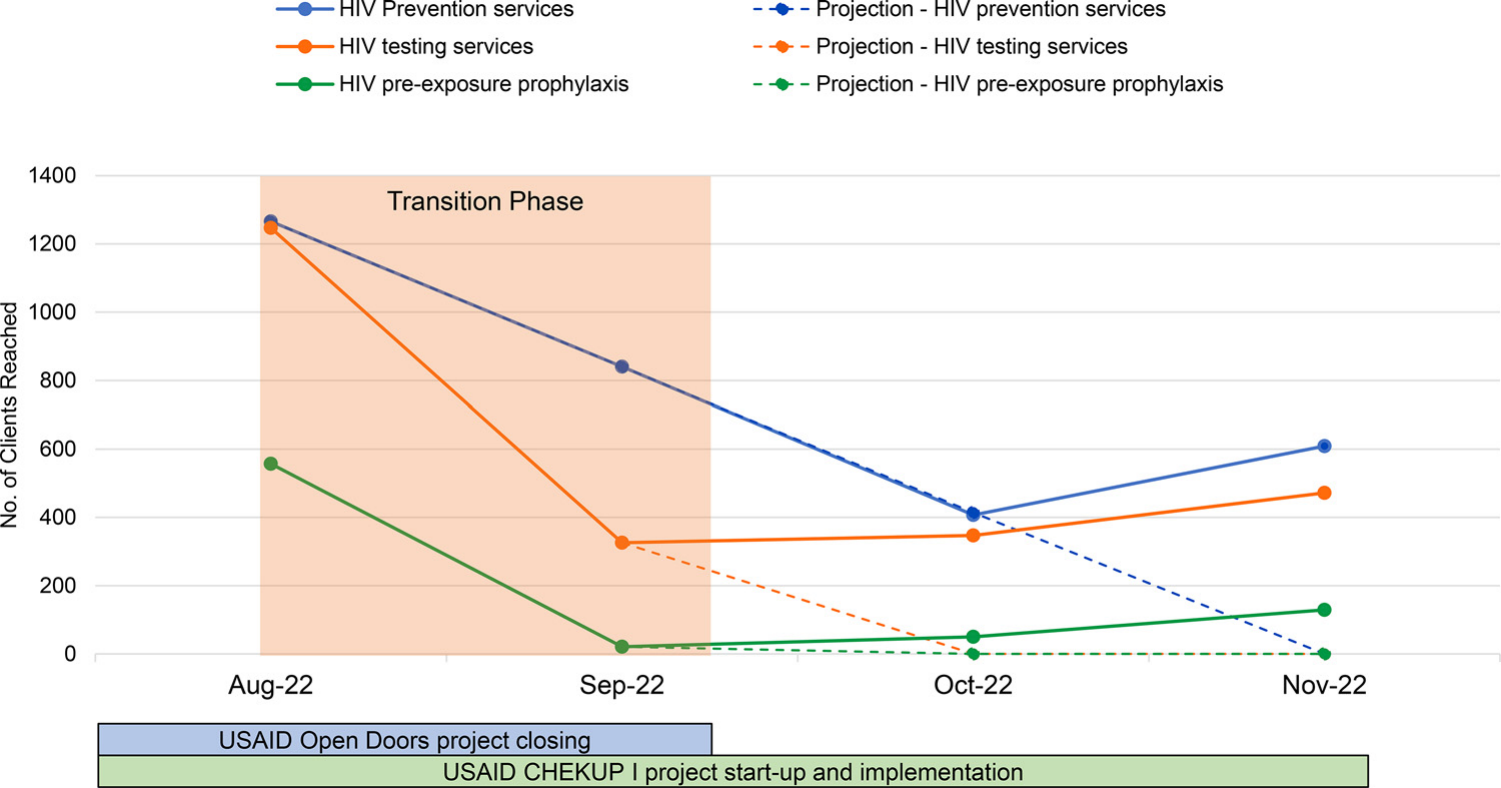
Actual and Projected HIV Service Uptake During Project Transition, Zambia, August to November 2022 September 2022 marked the point when USAID Open Doors (the closing project) ended service delivery and USAID CHEKUP I (the follow-on project) started providing services.

During the transition period, the same services continued in 3 districts (Solwezi, Chililabombwe, and Kitwe) for which both projects had responsibility. The timeline for service delivery and reporting was clearly split to avoid overlaps. USAID Open Doors supported service delivery until September 2022 and CHEKUP I thereafter. Figure 3 shows the trend in number of clients reached in these 3 districts with 3 types of services during the last 2 months for the closing project (August to September 2022) and the first 2 months for the new project (October to November 2022). Toward the end of the closing project, the service delivery trend for all 3 service types was a negative slope. It is important to note that the project was responsible for full service delivery during these months. From October to December 2022, the administrative closeout of the USAID Open Doors project occurred. The new project commenced KP activities in July 2022 and started service delivery in October 2022. After commencing service delivery, the new project immediately recorded a significant upward trend for all 3 service types, indicating that no start-up interruptions were experienced by the new project.

We assumed that the reach of clients through project efforts followed a linear progression. We then constructed a linear projection of clients that could have been reached if the new follow-on project (Figure 3) had not provided services, assuming there had been no coordinated transition. The projections show a drop to zero that may have happened within 2 months for all service delivery types. Instead, the new project reached 609 persons with HIV prevention services in November 2022, a 47% increase compared with those they reached in October 2022. Similarly, the new project reached 347 persons with HIV testing services in October 2022 (instead of the projected zero clients), which increased to 472 clients in November. Provision of PrEP to clients also followed a similar pattern, surpassing the reach in the closing months of USAID Open Doors.

## LESSONS LEARNED

### Meaningful Stakeholder Engagement Supports a Smooth Transition

The service delivery results show that with meaningful engagement of all key stakeholders, there can be uninterrupted service delivery during the transition process between projects and between international and local partners. This is a major benefit of active transition management as described by Huffstetler and colleagues.^29^ It was key to ensure that all key players, including the implementers and community representatives, supported the elements of the transition process.

### Early Planning of Transitions Contributes to Uninterrupted Service Delivery

The results show that services delivered to clients increased in the first 2 months of the new project compared to projections for that period. Some services were delivered to more clients in the first month of the new project than in the last month of the closing project. This is similar to the gains achieved through the coordination of clinical care that ensures continuity of care.^30^ Coordinating the project closeout and new project start-up, in our case, contributed to uninterrupted service delivery. This has been reported during other project transitions as well.^31^ Indeed, it has been argued that early planning of transitions is necessary for success.^11^ Although our experience shared in this paper predates the abrupt cessation of project activities following a US Government Stop Work Order issued in January 2025, the projected losses following that abrupt cessation of project activities^32^^,^^33^ further lend credence to the importance of lessons learned from our experience.

### Open Communication Fosters Trust and Cooperation

Open communication and well-meaning decisions facilitated trust and cooperation from the KP CSOs and other stakeholders. Both the outgoing and incoming project teams focused on the common goal of continuing service provision. The results observed were built on a foundation of trust and shared commitment to public good by all parties involved. The open communication around concerns and aims ensured that all parties had a shared vision, could trust each other, and were focused on achieving the goal of uninterrupted services. These elements have been described as critical to the success of interventions in different contexts.^29^^,^^34^^,^^35^

### Employing Closeout Project Staff Leverages Knowledge and Skills

It is common for staff on a closing project to apply for similar roles on a starting project. Managing these staffing transitions well had several benefits. First, it allowed minimal disruption to the existing project. The Open Doors team was notified when staff successfully interviewed for the CHEKUP I project, and their transition data was carefully negotiated to ensure that services were not disrupted on Open Doors while they transitioned to CHEKUP I. Resumption dates were agreed in consultation with the staff and both projects. USAID Open Doors project had ample notice to plan for coverage when the affected staff would be leaving. Second, it allowed the starting project to leverage years of USAID investment in building staff capacity on the closing project rather than building capacity from the beginning. Established relationships with community stakeholders could also be leveraged for the new project’s success. Careful review, selection, and interviews by the new project team prevented the transfer of staff who performed below par on the closing project. Staff were not transitioned to the new project based on mere recommendation or subjective selection but through a rigorous process that followed well-established policies and protocols.

### Maintaining Existing Service Delivery Locations Prioritizes Community Access

Often, when new projects start, they want to locate services in a new location to create a fresh identity for themselves. Unfortunately, this may lead to disorientation of clients within the communities who then wonder where to access services. This pitfall was avoided by the deliberate takeover of existing project locations within the communities. This was important as the choice of the locations was informed by clients.

### Donor Leadership Is a Critical Catalyst

The role of the funder is a critical catalyst in this transition process. With clear indication from the agreement officer representatives of both projects that uninterrupted services were a highly desired goal, the leadership of both projects galvanized action from their teams. Having the funder create the platform that brought both projects together was an essential point that set up a subsequent chain of actions. The initial planning meetings USAID staff convened were noted to be essential to the successful transition. Indeed, it has been established that transitions are influenced by the actions of donors as well as other players. This is also closely related to the role of leadership during transition, which has an important cascading effect that can help achieve transition goals.^29^

While the transition between the USAID Open Doors and USAID CHEKUP I projects demonstrated a model for the smooth transition between projects, was well managed, and led to good outcomes, we opine that there are broader considerations that contributed to these outcomes, especially in the pre-transition and post-transition phases. This transition model we discuss illustrates impactful synergy and collaboration between projects to provide a sustained way of service provision to clients. This approach can inform policy direction for future programming and leveraging resources. The funder realizes its vision of sustained local capacity-building toward a trajectory of sustained impact. There may be arguments against this model being donor driven rather than government led. Indeed, we acknowledge that this is our setting and note that the donor’s role here may be played by the government in other contexts as appropriate. This is particularly true in contexts where projects are being transitioned from donors to governments, as described by other authors.^36^

## RECOMMENDATIONS

Noting how critical project transitions are to ensuring continuous growth in project objectives, governments should require donors and implementers to describe specific transition phase management approaches in their overall project management plans. In addition, governments should hold implementers accountable for smooth project transitions. This active role would make the government more prominent in overall project transition management and help set the tone for the health system and the benchmarks for what is and is not acceptable.

From the proposal stage, donors should require implementers to describe specific transition phase management approaches in their overall project management plans when developing project designs. Donors for funded projects should actively lead the conversation around transitions and bring managers of closing and starting projects together in good time to start transition planning.

Finally, implementers and community project stakeholders should be aware of the transition phase, plan for potential challenges, and be active players in demanding and supporting a smooth transition. Implementers should ensure meaningful client inclusion and engagement as a key element to successfully transition services. All elements of project management, including community engagement, staffing, service delivery location, physical assets, and data management, should be intentionally managed for a successful transition.

## CONCLUSION

We believe that organizations should endeavor to openly communicate and engage each other so that clients are not negatively affected during project transitioning. Planning is key. Timely engagement of stakeholders will allow for all processes, including data transfer, asset transfer, and human resources, to be smoothly transitioned. The donor and government should foster this approach and build on it for implementation in all new and existing projects to ensure continuity of services to communities.

## Notes

**Peer Reviewed**

**First Published Online:** December 10, 2025.
